# Early treatment of imported falciparum malaria in the intermediate and intensive care unit setting: an 8-year single-center retrospective study

**DOI:** 10.1186/cc6796

**Published:** 2008-02-22

**Authors:** Lukas Schwake, Judith Pamela Streit, Lutz Edler, Jens Encke, Wolfgang Stremmel, Thomas Junghanss

**Affiliations:** 1 Department of Internal Medicine IV (Gastroenterology, Infectious Diseases and Intensive Care Medicine), University Hospital Heidelberg, Im Neuenheimer Feld, D-69120 Heidelberg, Germany; 2 Department of Biostatistics, German Cancer Research Center, Im Neuenheimer Feld, D-69120 Heidelberg, Germany; 3 Section of Clinical Tropical Medicine, University Hospital Heidelberg, Im Neuenheimer Feld, D-69120 Heidelberg, Germany

## Abstract

**Introduction:**

Imported falciparum malaria is characterized by a broad spectrum of potentially life-threatening complications that may arise even after initiation of appropriate antimalarial drug therapy. Hence, at Heidelberg University Hospital, all patients with newly diagnosed falciparum malaria are initially treated in the intermediate care unit (IMC) or intensive care unit (ICU). The present study was undertaken to evaluate critically the benefit of this strategy, which includes daily consultation with senior specialists in tropical medicine.

**Methods:**

We conducted a retrospective cohort study at the 14-bed combined IMC/ICU of a 1,685-bed university hospital. A cohort of 122 patients with imported falciparum malaria admitted from 1 January 1996 to 31 December 2003 was included.

**Results:**

Thirty-four patients (27.9%) developed complications, defined according to the current World Health Organization classification. Most patients (80.3%) studied did not take the recommended chemoprophylaxis against malaria. The majority of patients (89.3% [n = 109]) could be adequately treated in the IMC. Life-threatening complications requiring ICU support occurred in 13 patients (10.7%). All complications were successfully managed. Fifty-five patients (45.1%) fulfilling recently published criteria for outpatient treatment had an excellent therapeutic response and did not require ICU support.

**Conclusion:**

This retrospective evaluation demonstrated favourable therapeutic results in hospitalized patients with imported falciparum malaria. Both initial treatment in the medical IMC/ICU and close collaboration between intensivists and specialists in tropical medicine may improve disease outcome among affected patients. Prospective studies are needed to confirm these preliminary findings.

## Introduction

With 10,000 to 12,000 diagnoses per year in the European Union and 1,300 in the USA, imported malaria represents a growing challenge for health practitioners in the Western world [1,2]. In Germany, 800 to 1,000 cases of imported malaria are registered each year, two-thirds of which are caused by *Plasmodium falciparum*, the causative agent of falciparum malaria. Severe or complicated falciparum malaria is characterized by a broad spectrum of life-threatening complications, in particular acute respiratory distress syndrome, renal failure, circulatory shock and coma [3-8]. The course of the disease is often precipitous, requiring rapid diagnosis and initiation of both specific antimalarial chemotherapy and appropriate supportive care [5,9]. Despite improvements in intensive care management, the case fatality rate for complicated malaria in the intensive care unit (ICU) is still high and may reach up to 40% [9-12]. Unfavourable outcomes are at least partly attributable to delayed initiation of appropriate supportive care [13-15]. Early initiation of intensified supportive care, including ICU admission, has therefore been proposed by several investigators [2,10]. At the opposite end of the spectrum, ambulatory treatment appears to be a safe option in selected patients, as highlighted in recent publications [16,17]. Reliable criteria must be established so that patients may be assigned to the appropriate management strategy. At our institution, all patients with newly diagnosed falciparum malaria are initially referred to a combined medical intermediate care unit (IMC)/ISU, regardless of the presence or absence of complications. The present study was designed to evaluate critically the performance of this treatment strategy with respect to complications and outcome.

## Materials and methods

### Study population and design

We evaluated all patients with imported falciparum malaria admitted to the combined medical IMC/ICU of Heidelberg University Hospital, Heidelberg, Germany, between 1 January 1996 and 31 December 2003, in a single-centre retrospective observational study. Inclusion criteria were as follows: minimum age 15 years and a newly diagnosed *P. falciparum *infection acquired in an endemic country and imported to Germany. In all cases the diagnosis of falciparum malaria was based on positive Giemsa-stained thin and thick blood smears, examined by experienced physicians of either the Department of Laboratory Medicine or the Section of Clinical Tropical Medicine. Ethics approval was obtained from the local ethics committee (Medical University Heidelberg).

### Setting

The 14-bed medical ICU at Heidelberg University Hospital is both a primary care and a tertiary referral centre for critically ill patients. The unit is split into a seven-bed, fully equiped ICU and a seven-bed intermediate care area (IMC). All patients are treated by the same physician and nursing team.

### Data collection

Patients' medical charts were reviewed retrospectively and the following data were collected: demographic baseline data and travel-related history (age, sex, region of travel, use of antimalarial chemoprophylaxis), latency period (time from arrival in Germany to presentation at hospital), signs and symptoms at presentation, laboratory results, treatment, complications and outcome data (mortality and length of stay both in the hospital and in the ICU). Disease severity on admission to the ICU or IMC was assessed by means of the Simplified Acute Physiology Score II [18]. Chemoprophylaxis was considered inappropriate when it was either not taken at all or inadequate. European travellers and tourists were considered nonimmune. Patients who were born and grew up in malaria endemic areas were defined as semi-immune.

### Patient management

Since 1996 all patients with microscopically confirmed falciparum malaria have been directly referred to the medical IMC/ICU. This treatment strategy was implemented after one fatal course during the preceding year and was intended to promote optimal monitoring and treatment conditions. After the initial assessment by the physician in charge, patients were either transferred to the IMC area or to a regular, fully equipped ICU bed. All patients received antimalarial drug therapy immediately and were continuously monitored. Basic and advanced supportive care measures provided to the patients are summarized in Table 5 (see below). Additionally, urine output was monitored hourly in critically ill patients and twice daily in uncomplicated cases. Administration of blood components was performed according to clinical judgement. Decisions regarding transfusion of red blood cells were based on published recommendations (fall of haemoglobin concentration by 20% within 24 hours or cardiac decompensation) [19]. None of the patients developed severe anaemia (haemoglobin <5 g/dl). Haemodialysis treatment was initiated in case of apparent renal failure, as defined by World Health Organization (WHO) criteria (Table 3; see below), despite adequate intravenous fluid replacement. Daily consultations with senior specialists in tropical medicine were held to discuss both antimalarial drug therapy and supportive care. Discharge from the IMC/ICU was based on consideration of the general status and clinical course of the patient.

**Table 3:** Complications

Criteria	Definition	Number of patients (%)
Unrousable coma	Glasgow Coma Scale score ≤9; exclude other causes; should persist ≥30 minutes after a generalized convulsion	3 (2,5)
Impaired consciousness	Mental clouding, rousable	11 (9)
Multiple seizures	Three or more convulsions observed within 24 hours	0 (0)
Respiratory distress	Acidotic breathing, pulmonary oedema, or acute respiratory distress syndrome, on the basis of radiographic densities, hypoxaemia, and positive end-expiratory pressure	4 (3.3)
Circulatory collapse or shock	Systolic blood pressure <80 mmHg despite adequate volume repletion	5 (4.1)
Abnormal bleeding	Spontaneous bleeding from gums, nose, gastrointestinal tract, or laboratory evidence of disseminated intravascular coagulation	4 (3.3)
Jaundice/hyperbilirubinaemia	Icteric sclera/buccal mucosa, or serum bilirubin >50 μmol/l (>3 mg/dl)	17 (13.9)
Severe anaemia	Heemoglobin concentration <5 g/l, or haematocrit <15%	0 (0)
Hypoglycaemia	Whole blood glucose concentration <2.2 mmol/l (<40 mg/dl)	3 (2.5)
Renal failure	Serum creatinine >265 μmol/l, oliguria (<400 ml/24 hours) despite adequate rehydration	5 (4.1)
Hyperparasitaemia	More than 5% parasitized erythrocytes or >250,000 parasites/μl (in nonimmune individuals)	13 (10.7)
Acidosis	Plasma bicarbonate <15 mmol/l or base excess under -10, or acidaemia (arteria/capillaryl pH <7.25)	4 (3.3)
Macroscopic haemoglobinuria	Dark or red urine; exclude haematuria, haemolysis not secondary to glucose-6-phosphate dehydrogenase deficiency	ND

The complications are defined according to the World Health Organization classification system [20] (n = 34 patients). ND, not determined

**Table 5:** Monitoring and supportive care procedures in all 122 patients

	Number of patients (%)
Monitoring and basic supportive care	122 (100)
Continuous monitoring (ECG, oxygen status, blood pressure)	122 (100)
Intravenous fluid and electrolyte replacement	86 (70.5)
Oxygen supply	32 (26.2)
Central venous catheterization	37 (30.3)
Arterial catheterization	9 (7.4)
Advanced supportive care	27 (22.1)
Transfusion of blood products	20 (16.4)
Inotropic drug therapy	5 (4.1)
Noninvasive ventilatory support	4 (3.3)
Invasive ventilation	3 (2.5)
Renal replacement therapy	4 (3.3)
Total parenteral nutrition	12 (9.8)

ECG, electrocardiogram.

### Drug treatment

Patients were treated with standard drug regimens, including quinine and doxycycline, atovaquone/proguanil, and mefloquine, bearing in mind drug resistance patterns, pretreatment, chemoprophylaxis taken and other individual factors (such as individual contraindications to a specific drug). Patients with complicated falciparum malaria received intravenous quinine with a loading dose on ICU admission. Patients without complications received intravenous quinine only if clinically indicated. Dose adjustments in patients with acute renal failure or hepatic dysfunction were performed as recommended by the WHO guidelines [19,20]. None of the patients originated from a multidrug-resistant region.

### Definition of complications

Serious or complicated malaria was defined according to the WHO criteria by the presence of one or more of the clinical and laboratory manifestations enlisted in Table 3 (see below), along with asexual forms of *P. falciparum *in peripheral blood smear [9,19-22]. The presence of haemoglobinuria could not be determined retrospectively. Serious complications requiring immediate medical intervention were defined as those that were life-threatening; they included circulatory shock, renal failure, respiratory failure, acidosis, abnormal bleeding, severe anaemia, coma or seizures, and hypoglycaemia.

### Data handling and statistical analysis

All patient data were documented on a spreadsheet (Excel 2000; Microsoft Corp, Redmond, WA, USA) and analyzed statistically using WinStat (R. Fitch Software, Staufen, Germany) and SPSS 13.0 (SPSS Inc., Chicago, IL, USA) software. Patients were categorized into groups on the basis of the presence or absence of complications. The distributions of continuous variables were checked for normality using the Kolmogorov–Smirnov test and, if appropriate, expressed as mean ± standard deviation. For some laboratory data, logarithmic transformation was applied to achieve normality. Continuous variables deviating from normality were presented as median with interquartile range (25th to 75th percentile). Unpaired Student's *t*-tests and Mann-Whitney U-test were used to compare two groups. Candidate clinical and laboratory predictors of complications that were statistically significant at the explorative level of 10% (*P *< 0.1) in the univariate analysis were subjected to binary logistic regression to determine the independent contribution of each possible risk factor. Receiver-operating characteristic curves and areas under the respective curve (AUCs) were further calculated, and model fit was assessed using the Hosmer-Lemeshow goodness-of-fit statistic [23]. Categorical data were presented as absolute numbers with associated percentages and evaluated using χ^2 ^or Fisher's exact test, as appropriate. All tests were applied two-tailed, and a *P *value of < 0.05 was considered to be statistically significant.

## Results

### Patient characteristics

**Figure 1:**
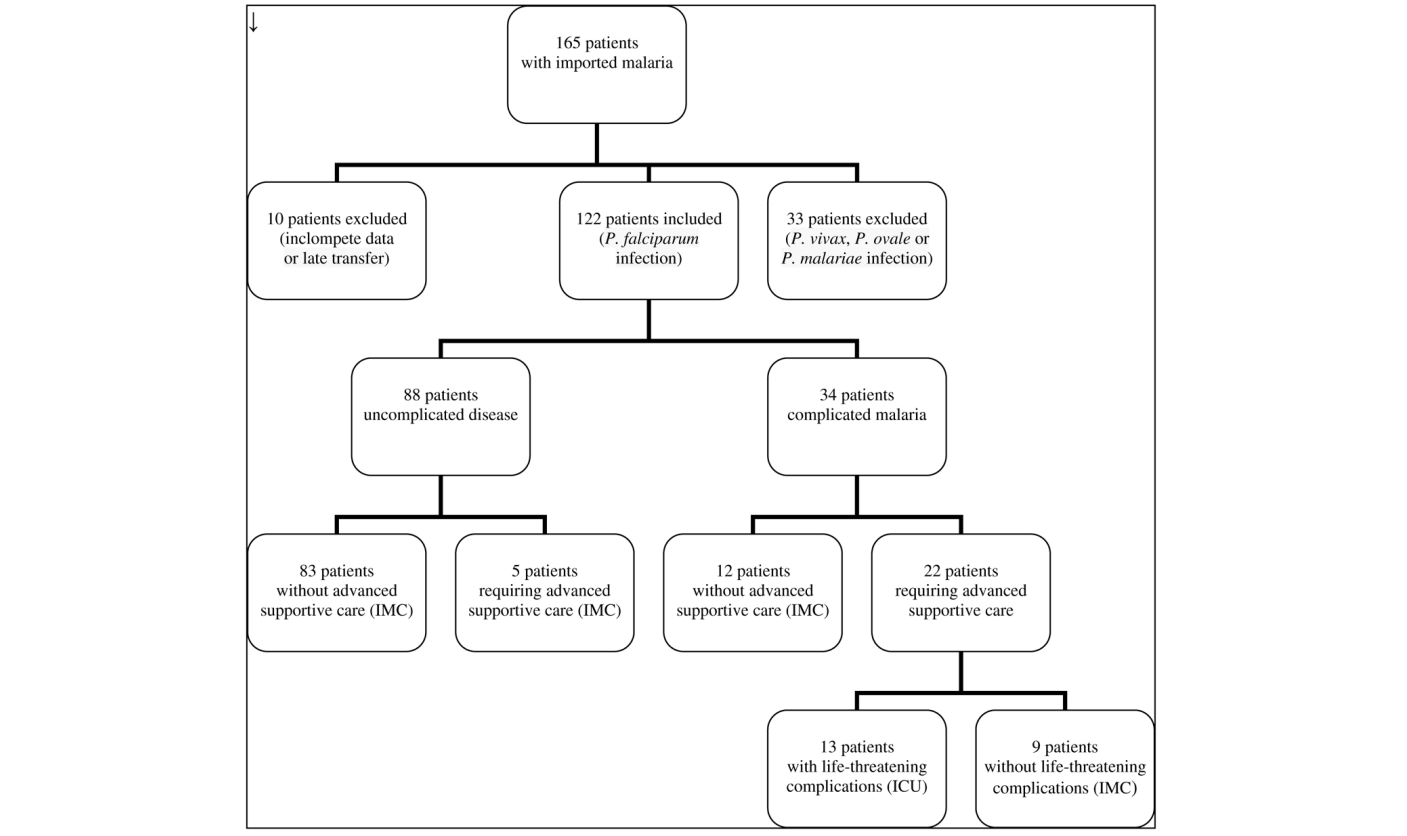
Flow chart of patients included in the study. ICU = intensive care unit; IMC = intermediate care unit.

Between January 1996 and December 2003, a total of 165 patients with malaria were admitted to the medical IMC/ICU. Of these patients, 122 with *P. falciparum *malaria were included in the present study. Thirty-three patients were excluded because of infection with other *Plasmodium *spp. (*P. vivax*, *P. ovale *and *P. malariae*) and ten patients because of late transfer (>24 hours after diagnosis) or incomplete data (Figure 1). A total of 116 patients were directly admitted to the IMC/ICU via the Medical Emergency Department or the outpatient clinic of the Section of Clinical Tropical Medicine. Six patients were transferred to the ICU from referral hospitals within 24 hours or less after diagnosis. The study population was predominantly male. During the 8-year study period there were no fatal cases of falciparum malaria. Table 1 lists patient characteristics, demographic profiles, and signs and symptoms at the time of admission to the IMC/ICU. Patients with complications were significantly older than patients without complications. All but 15 of the 122 patients acquired *P. falciparum *malaria in sub-Saharan Africa. Other regions of travel were Southern Africa, Asia and tropical South America, accounting for eight cases, six cases and one case, respectively. Chemoprophylaxis was inappropriate in 98 patients (80.3%); it was not taken at all by 74 patients and inadequately by 24 patients.

**Table 1:** Baseline characteristics at admission to the ICU

	All malaria patients (n = 122 [100%])	Noncomplicated malaria (n = 88 [100%])	Complicated malaria (n = 34 [100%])	*P*
Demographics				
Age (years; mean ± SD)	38.4 ± 11.1	37.1 ± 10.2	42 ± 12.8	0.049
Male sex (*n *[%])	88 (72.1)	62 (70.5)	26 (76.5)	0.506
Return from Sub-Saharan Africa (*n *[%])	107 (87.7)	76 (86,4)	31 (91.2)	0.350
Nonimmune patients (*n *[%])	88 (72.1)	62 (70.5)	26 (76.5)	0.506
Compliant chemoprophylaxis users (*n *[%])	24 (19.7)	17 (19.3)	7 (20.6)	0.874
Latency period (arrival-admission; days; median [IQR])	10 (6–15)	10 (7–15)	8 (4–14)	0.778
Initial signs and symptoms				
Fever (>37°C; *n *[%])	118 (96.7)	84 (95.5)	34 (100)	0.265
Headache (*n *[%])	71 (58.2)	57 (64.8)	14 (41.2)	0.018
Chills (*n *[%])	43 (35.2)	31 (35.2)	12 (35.3)	0.994
Diarrhoea (*n *[%])	46 (37.7)	29 (33)	17 (50)	0.082
Nausea (*n *[%])	47 (38.5)	31 (35.2)	16 (47.1)	0.229
Vomiting (*n *[%])	29 (23.8)	20 (22.7)	9 (26.5)	0.687
Jaundice (*n *[%])	11 (9)	0 (0)	11 (32.4)	0.000
Confusion (*n *[%])	5 (4.1)	0 (0)	5 (14.7)	0.001
Vertigo (*n *[%])	25 (20.5)	14 (15.9)	11 (32.4)	0.044
Myalgias (*n *[%])	47 (38.5)	34 (38.6)	13 (38.2)	0.967
Abdominal pain (*n *[%])	10 (8.2)	6 (6.8)	4 (11.8)	0.893
Simplified Acute Physiology Score II (median [IQR])	13 (9–20)	13 (9–17)	27 (17–36)	0.000
IMC/ICU length of stay (days; median [IQR])	5 (3–6)	4 (2–5)	6 (5–8)	0.000
Hospital length of stay (days; median [IQR])	6 (5–8)	5.5 (4–7)	8 (7–10)	0.000

ICU, intensive care unit; IMC, intermediate care unit; IQR, interquartile range (25th to 75th percentile); SD, standard deviation.

Fifty-five patients (45.1%) fulfilled recently published criteria for ambulatory treatment, namely parasitaemia under 1%, absence of complications, age 16 years or older, and no vomiting [17]. In one of these patients hyperbilirubinaemia was observed. None of these 55 patients required advanced supportive care.

### Laboratory results

Haematological and biochemical findings on admission are shown in Table 2. Univariate logistic regression detected significant differences between complicated and uncomplicated malaria in 12 of the 16 parameters tested. Serum bilirubin, lactate dehydrogenase and platelet concentrations exhibited the greatest differences between groups. Significant differences were also observed for initial and peak parasitaemia (*P *< 0.0001); median parasitaemia on admission was 0.7% (interquartile range 0.1% to 5%) for uncomplicated cases, and 21% (2.5% to 45%) for patients with complications defined according to WHO criteria. Parasitaemia increased to a median of 1.6% (0.2% to 5.7%) in patients without complications and 34% (11.5% to 72.1%) in complicated malaria.

**Table 2:** Laboratory findings at admission to the ICU

Parameter	All malaria patients (n = 122 [100%])	Noncomplicated malaria (n = 88 [100%])	Complicated malaria (n = 34 [100%])	*P*	Numbers of patients tested
Haemoglobin (g/dl; mean ± SD)	13.7 ± 2	14 ± 1.9	12.9 ± 2.3	0.007	122
Platelet count (× 10^9^/l; mean ± SD)	89 ± 51	104 ± 49	50 ± 32	0.000	122
White blood cell count (× 10^9^/l; mean ± SD)	5.4 ± 2.2	5.1 ± 2.0	6.1 ± 2.5	0.023	122
Plasma prothrombin time (%; mean ± SD)	78 ± 17	79 ± 16	73 ± 17	0.066	122
Serum sodium (mmol/l; mean ± SD)	134 ± 4.7	135 ± 4.2	131 ± 4.6	0.000	122
Serum potassium (mmol/l; mean ± SD)	3.9 ± 0.5	3.8 ± 0.4	3.9 ± 0.7	0.739	122
Serum glucose (mg/dl; mean ± SD)	119 ± 32	124 ± 38	117 ± 29	0.287	122
Total bilirubin (mg/dl; mean ± SD)	1.5 ± 1.8	0.9 ± 0.4	2.8 ± 2.7	0.000	98
C-reactive protein (mg/l; mean ± SD)	102 ± 63	82 ± 45	151 ± 73	0.000	113
Serum aspartate aminotransferase (U/l, median [IQR])	37 (27–72)	33 (26–59)	61 (35–88)	0.001	122
Serum alanine aminotransferase (U/l; mean ± SD)	60 ± 39	56 ± 36	72 ± 44	0.045	122
Serum creatinine (mg/dl; median [IQR])	1.1 (0.9–1.3)	1 (0.9–1.2)	1.2 (1.1–1.8)	0.000	122
Serum urea (mg/dl [mean ± SD])	40 ± 32	29 ± 11	67 ± 48	0.000	122
Serum alkaline phosphatase (U/l [mean ± SD])	83 ± 36	81 ± 31	88 ± 46	0.457	96
γ-Glutamyl transferase (U/l; mean ± SD)	68 ± 89	60 ± 92	85 ± 79	0.213	99
Lactate dehydrogenase (U/l; mean ± SD)	333 ± 202	259 ± 81	524 ± 282	0.000	122

ICU, intensive care unit; IMC, intermediate care unit; IQR, interquartile range (25th to 75th percentile); SD, standard deviation.

Mild hyponatraemia (serum sodium 125 to 135 mmol/l) at admission was a common finding and occurred in 77 patients (63.1%). However, only one patient exhibited a serum sodium concentration below 124 mmol/l. In addition, an increase in aspartate aminotransferase to more than three times the upper normal limit – usually regarded 'malaria hepatitis' – was documented in 27 patients (22.1%).

### Complications

Table 3 shows the complications recorded on admission and occurring during the IMC/ICU stay. Twenty-one of the 34 patients with a complicated course exhibited signs of complications at the time of admission to the IMC/ICU, whereas another 13 patients without initial complications developed them during their stay in the IMC/ICU. Hyperparasitaemia (> 5% parasitized erythrocytes) was present in eight patients on admission and developed in five further patients during their hospital stay. The corresponding numbers for hyperparasitemia above 10% were five and one. Twenty-one patients developed one complication, six patients two complications and seven patients three or more complications. Life-threatening complications requiring ICU support were observed in seven patients at admission and developed in six further patients during the IMC/ICU stay. Three of the latter did not exhibit any complications at the time of admission. The time course of life-threatening complications is shown in Table 4.

**Table 4:** Life-threatening complications in 13 patients with imported falciparum malaria

Complication	Present on admission (*n*)	Developing during ICU stay (*n*)
Unrousable coma	0	3
Respiratory distress	2	2
Circulatory collapse or shock	0	5
Abnormal bleeding/disseminated intravascular coagulation	4	0
Hypoglycaemia	0	3
Acidosis	2	2
Renal failure	2	3
Multiple seizures	0	0
Severe anaemia	0	0

ICU, intensive care unit.

Four of the five patients fulfilling the criteria for acute renal failure required haemodialysis. In one patient with renal failure, haemodialysis could be avoided by careful intravenous rehydration. Parasitaemia and markers of haemolysis (lactate dehydrogenase, aspartate aminotransferase and bilirubin) were significantly higher in patients with acute renal failure than in those with other complications, both on admission and during hospital stay (data not shown).

### Treatment

Quinine was given to 56 patients (45.9%), 44 of whom received it intravenously. In 21 patients (17.2%) quinine was given in combination with oral doxycyclin. Thirty-four patients (27.9%) received mefloquine. Atovaquone/proguanil was given in 42 cases (34.4%), as an exclusive treatment in 32 patients and to complete treatment after initial quinine therapy in 10. A total of 270 blood units, including 78 packed red blood cells, 82 thrombocyte concentrates and 110 fresh frozen plasma, were transfused in 20 patients. Basic and advanced supportive care measures for the 122 study patients are listed in Table 5. Empirical antibiotic treatment was given in four patients with either persistent shock or probable pneumonia. All patients recovered completely and were discharged from the hospital without residual sequelae.

### Prognostic factors

**Figure 2:**
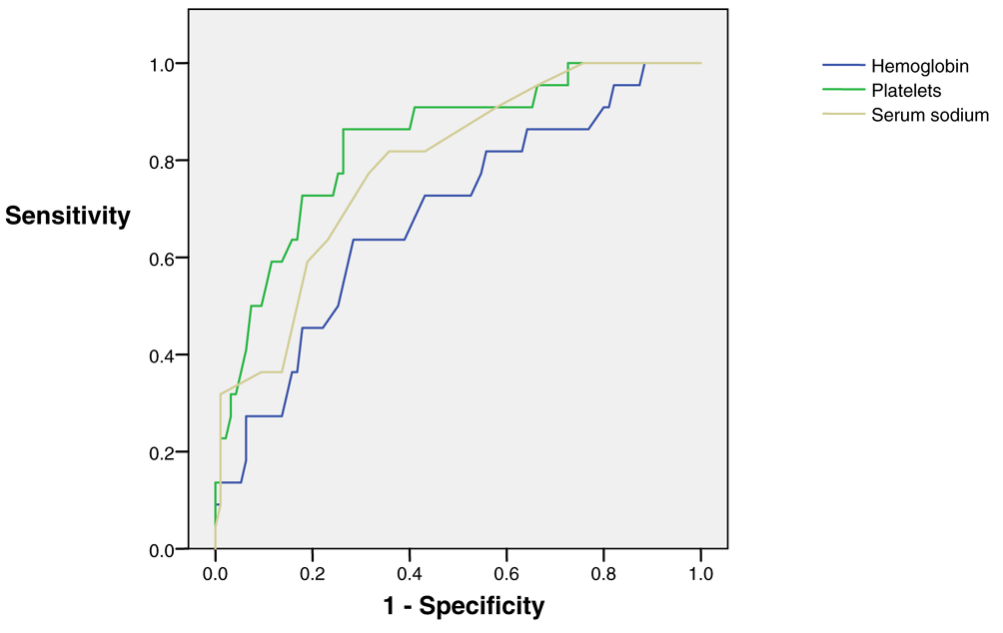
ROC curve analysis of three statistically significant predictors of need for advanced supportive care. A total of 108 patients with imported falciparum malaria were included in the analysis. ROC, receiver operating characteristic.

Significant and nearly significant (*P *< 0.1) variables from the univariate analysis were included in a multivariate binary logistic regression model. Because of the small number of patients with secondary acquisition of complications, we opted to choose the need for advanced supportive care (Table 5) as the variable of interest. Five patients needed immediate advanced supportive care at the time of admission and were therefore excluded from this analysis. A further nine patients (including one patient with advanced supportive care) were excluded because of missing laboratory data from chart review. The Hosmer-Lemeshow goodness-of-fit χ^2 ^statistic was 9.548, with eight degrees of freedom (*P *= 0.298), which indicates excellent calibration of the model. Binary logistic regression demonstrated that haemoglobin and platelet levels as well as serum sodium concentration at admission were inversely associated with the need for advanced supportive care (Table 6). The AUC for blood platelets was 0.837 (standard error 0.047; *P *< 0.001; 95% confidence interval 0.744 to 0.929), suggesting good accuracy of blood platelets in predicting the need for advanced supportive care in the ICU. For comparison, sodium levels had an AUC of 0.789 (standard error 0.051; *P *< 0.001; 95% confidence interval 0.690 to 0.889) and blood haemoglobin concentration had an AUC of 0.686 (standard error 0.064; *P *= 0.007; 95% confidence interval 0.561 to 0.811), indicating that these variables has less discriminative power (see Figure 2).

**Table 6:** Predictors of advanced supportive care in multivariate logistic regression analysis

Variable	Coefficient	Standard error	Odds ratio	95% confidence interval	*P*
Haemoglobin (g/dl; mean ± SD)	-0.374	0.174	0.688	0.489–0.967	0.031
Platelet count (× 10^9^/l, mean ± SD)	-0.032	0.012	0.968	0.946–0.991	0.007
Serum sodium (mmol/l; mean ± SD)	-0.213	0.085	0.808	0.685–0.954	0.012

A total of 108 patients were included in this analysis. SD, standard deviation.

## Discussion

Because of the dynamics of *P. falciparum *infection, patients may deteriorate within 24 hours despite initiation of adequate antimalarial treatment [19,24]. Thus far, however, reliable criteria for ICU admission as well as standardized protocols for the supportive treatment of complications have not been established [2,10]. This retrospective cohort study describes clinical features and treatment of 122 patients with imported falciparum malaria admitted to the combined medical IMC/ICU of a university hospital over an 8-year period. In contrast to conventional practice, all patients with newly diagnosed *P. falciparum *infection were initally referred to the IMC/ICU, regardless of the presence or absence of complications. Our results clearly show that early IMC/ICU treatment of all patients with imported falciparum malaria is associated with a favourable clinical outcome; none of the patients died and all were discharged from the hospital without sequelae.

Previous studies of the treatment of imported malaria in the ICU have reported much higher mortality rates. In a study reported in 2003 [2], a French group evaluated 188 patients admitted to an ICU for treatment of imported falciparum malaria over a 12-year period. In contrast to our patient cohort, all patients included in that study suffered from complicated malaria and their overall mortality was 5.3%. A few years earlier the same authors described 50 patients with complicated falciparum malaria treated in a medical ICU over a 6-year period [25]. The corresponding mortality rate was 14% in this analysis, mostly due to refractory circulatory shock. In a survey from Austria [10] seven out of 69 patients with falciparum malaria were admitted to the medical ICU for treatment of complications; three patients died despite sophisticated ICU management, corresponding to an ICU mortality rate of 40%. In a recently published analysis from Germany [26], 21 out of 116 patients with falciparum malaria were treated in the ICU, of whom two patients died.

In all of these studies patients were only transferred to the ICU in case of serious complications or deterioration, despite appropriate antimalarial drug therapy. This strategy, which reflects usual clinical practice, has several pitfalls, however. Doctors and nursing staff on regular (non-ICU) wards may face problems in providing the 24-hour monitoring that is necessary to detect arising complications rapidly. Furthermore, loss of information during ICU transfer may cause delayed and suboptimal treatment. Our approach differs markedly from this 'traditional' treatment policy, mainly by providing a higher level of initial observation and by shortening the response to upcoming complications. Indeed, this strategy enabled us to successfully manage all arising complications over an 8-year period. We hypothesize that a number of potentially preventative measures in the IMC contributed to the low rate of major complications in our patient cohort: intense monitoring, close surveillance by doctors and nursing staff, and rapid medical response to upcoming complications.

As shown by others, rapid correction of both hypovolaemia and circulatory overload by means of intravenous hydration and diuretic therapy is essential to prevent the development of life-threatening complications, including circulatory shock, acute renal failure and acute respiratory distress syndrome [19]. Early transfusion of blood products (none of the patients developed severe anaemia) might have further supported these effects. Acute renal failure that is (as in our patient cohort) typically associated with high parasitaemia and significant haemolysis have been shown to carry particular risk [27,28]. Hence, in our patient group renal replacement therapy was promptly initiated once signs of acute renal failure were present and did not rapidly improve with intravenous rehydration. This strategy is consistent with suggestions by other authors and has been shown to be beneficial [27,29,30].

Our study raises additional concerns that must be addressed. The data presented herein clearly show that initial IMC/ICU admission does not completely prevent the development of serious complications; life-threatening complications requiring immediate intervention developed in six patients after initiation of treatment in the IMC/ICU. Most importantly, half of these patients did not have any complications at the time of admission. One explanation for this phenomenon is that side effects of antimalarial drugs as well as nosocomial infections may facilitate the development of serious complications [8,24]. In two of the three patients mentioned above, severe hypoglycaemia, probably due to quinine administration and the parasites' glucose demand, was the only complication observed during the ICU stay. Another four patients exhibited clinical signs of septicaemia requiring broad-spectrum antibiotic treatment. These observations illustrate both the difficulties in predicting severe courses of the disease and the need to monitor treatment complications.

Initial treatment of all patients with imported falciparum malaria in the hospital setting continues to be the most common practice in Western countries [24]. However, results from two recently published prospective observational studies have shown that outpatient treatment of uncomplicated falciparum malaria in adults with low parasitaemia (< 1%) and without vomiting can be safe and effective [16,17,31]. Although our study was not initially designed to address this issue, our findings in a subgroup analysis support these findings.

By using binary logistic regression, we identified thrombocytopenia, hyponatraemia and anaemia to be of decreased significance as predictors of need for advanced supportive care. As with anaemia, low platelets have been identified as markers of severity and predictors of fatality by other investigators [2,11,27-30]. In contrast, hyponatraemia in falciparum malaria is usually mild and not regarded to be an indicator of complications. In rare cases, however, severe hyponatraemia may cause cerebral oedema and coma, and thus complicated falciparum malaria [31,32]. In view of these findings it is noteworthy that factors predicting disease severity vary between study centres and cannot be generalized for all conditions [11,29,32-34]. Hence, the presence of thrombocytopenia, hyponatraemia and/or anaemia at admission should raise particular concern about secondary complications requiring advanced supportive care. Their absence, however, does not exclude fulminate courses of the disease. Also, because of the retrospective nature of the study, neither plasma lactate at admission nor time from symptom onset to antimalarial therapy initiation could be retrieved from the patients' charts. Both parameters have been shown to be important prognostic factors in falciparum malaria [35].

An important finding of our study is that the vast majority (80.3%) of our patients took either no or inappropriate chemoprophylaxis. This is comparable to the findings of other studies and proves once again the need for more effective prevention strategies, including better patient complicance [2,36,37].

Our study has limitations that should be mentioned. First, as a retrospective case review performed in a single centre, it is subject to several possible biases, namely reporting bias and selection bias. Hence, because of its retrospective nature, our evaluation can generate hypothesis but cannot prove causality. In particular, we cannot definitely exclude the possibility that the low number of serious complications and the favourable therapeutic outcomes in our patient group is due to selection rather than to treatment strategy. Second, other centres have reported low or no mortality even without early IMC/ICU treatment [37-39]. Hence, other factors, including rapidity and accuracy of diagnosis, but also the multidisciplinary nature and availability of specialists in German tropical medicine departments (who are usually affilitated to university hospitals), should also be considered as important determinants of treatment response. Third, we did not have a control group of patients treated in accordance with conventional practice. However, even in a prospective design, ethical considerations would probably have prevented us from including one. Finally, additional treatment costs as well as appropriate treatment duration in the IMC/ICU remain to be determined. In times of rationing ICU resources, this question is of particular importance. In the future, alternatives to our strategy may arise from the implementation of rapid response systems, including in-hospital medical emergency teams [40,41].

## Conclusion

In summary, this is the first study to show that early monitoring and treatment in the IMC/ICU is indeed associated with a favourable therapeutic outcome in patients with imported falciparum malaria. However, because of methodological limitations we cannot recommend a general change in common treatment policy.

## Key messages

• Hospitalized patients with imported falciparum malaria may benefit from early treatment in the IMC/ICU.

• During therapy it is important to monitor for treatment complications and nosocomial infections.

• Further studies with improved design are necessary to confirm our findings.

## Abbreviations

ICU = intensive care unit; IMC = intermediate care unit; WHO = World Health Organization.

## Competing interests

The authors declare that they have no competing interests.

## Authors' contributions

LS designed the study, carried out the statistical analysis and wrote the manuscript. JPS performed data acquisition and contributed to the design and development of the report. LE contributed to the statistical analysis and interpretation of the results. JE and WS supervised LS and JPS and contributed to data interpretation. TJ co-wrote the first draft of the manuscript and contributed to data analysis and interpretation. All authors read and approved the final manuscript.

## References

[B1] Jelinek T, Schulte C, Behrens R, Grobusch MP, Coulaud JP, Bisoffi Z, Matteelli A, Clerinx J, Corachan M, Puente S, Gjorup I, Harms G, Kollaritsch H, Kotlowski A, Bjorkmann A, Delmont JP, Knobloch J, Nielsen LN, Cuadros J, Hatz C, Beran J, Schmid ML, Schulze M, Lopez-Velez R, Fleischer K, Kapaun A, McWhinney P, Kern P, Atougia J, Fry G (2002). Imported falciparum malaria in Europe: sentinel surveillance data from the European network on surveillance of imported infectious diseases. Clin Infect Dis.

[B2] Bruneel F, Hocqueloux L, Alberti C, Wolff M, Chevret S, Bedos JP, Durand R, Le Bras J, Regnier B, Vachon F (2003). The clinical spectrum of severe imported falciparum malaria in the intensive care unit. Am J Respir Crit Care.

[B3] Kain KC, Harrington MA, Tennyson S, Keystone JS (1998). Imported malaria: prospective analysis of problems in diagnosis and management. Clin Infect Dis.

[B4] Gregorakos L, Sakayianni K, Hroni D, Harizopoulou V, Georgiadou F, Adamidou M (1999). Management of severe and complicated malaria in the intensive care unit. Intensive Care Med.

[B5] Patel DN, Pradeep P, Surti MM, Agarwal SB (2003). Clinical manifestations of complicated malaria: an overview. J Indian Assoc Child Adolesc Ment Health.

[B6] Trampuz A, Jereb M, Muzlovic I, Prabhu RM (2003). Clinical review: severe malaria. Crit Care.

[B7] Schwake L, Junghanss T, Weimann J, Stremmel W (2001). Imported tropical malaria after a sojourn in Kenya. Serious consequences of neglected chemoprophylaxis and delayed diagnosis. Dtsch Med Wochenschr.

[B8] Gachot B, Wolff M, Nissack G, Veber B, Vachon F (1995). Acute lung injury complicating imported Plasmodium falciparum malaria. Chest.

[B9] The World Health Organization Guidelines for the Treatment of Malaria.

[B10] Losert H, Schmid K, Wilfing A (2000). Experiences with severe *P. falciparum *malaria in the intensive care unit. Intensive Care Med.

[B11] Krishnan A, Karnad DR (2003). Severe falciparum malaria: an important cause of multiple organ failure in Indian intensive care unit patients. Crit Care Med.

[B12] Koh KH, Chew PH, Kiyu A (2004). A retrospective study of malaria infections in an intensive care unit of a general hospital in Malaysia. Singapore Med J.

[B13] Newman RD, Parise ME, Barber AM, Steketee RW (2004). Malaria-related deaths among U.S. travellers, 1963–2001. Ann Intern Med.

[B14] Backmund M, Von Zielonka M, Hartmann WJ, Hesse J, Eichenlaub D (1999). Malaria: state of the art. I. Epidemiology, forms of malaria, diagnosis. Fortschr Med.

[B15] Püschel K, Lockemann U, Dietrich M (1998). Malaria-immer wieder Todesfälle infolge verspäteter Diagnose [in German]. Dt Aerzteblatt.

[B16] D'Acremont V, Landry P, Darioli R, Stuerchler D, Pecoud A, Genton B (2002). Treatment of imported malaria in an ambulatory setting: prospective study. BMJ.

[B17] Bottieau E, Clerinx J, Colebunders R, Van den Enden E, Wouters R, Demey H, Van Esbroeck M, Vervoort T, Van Gompel A, Van den Ende J (2006). Selective ambulatory management of imported falciparum malaria: a 5-year prospective study. Eur J Clin Microbiol Infect Dis.

[B18] Le Gall JR, Lemeshow S, Saulnier F (1993). A new acute simplified score (SAPS II) based on a European/North American multicenter study. JAMA.

[B19] Mishra SK, Mohanty S, Mohanty A, Das BS (2006). Management of severe and complicated malaria. J Postgrad Med.

[B20] The World Health Organization (WHO) (2000). Severe falciparum malaria, severe and complicated malaria. Trans R Soc Trop Med Hyg.

[B21] The World Health Organization (WHO) (1990). Severe and complicated malaria. Trans R Soc Trop Med Hyg.

[B22] Planche T, Krishna S (2005). The relevance of malaria pathophysiology to strategies of clinical management. Curr Opin Infect Dis.

[B23] Concato J, Feinstein AR, Holford TR (1993). The risk of determining risk with multivariable models. Ann Intern Med.

[B24] Whitty CJM, Lalloo D, Ustianowski A (2006). Malaria: an update on treatment of adults in non-endemic countries. BMJ.

[B25] Bruneel F, Gachot B, Timsit JF, Wolff M, Bedos JP, Regnier B, Vachon F (1997). Shock complicating severe falciparum malaria in European adults. Intensive Care Med.

[B26] Rabe C, Paar WD, Knopp A, Munch J, Musch A, Rockstroh J, Martin S, Sauerbruch T, Dumoulin FL (2005). Malaria in the emergency room. Results of the emergency treatment of 137 patients with symptomatic malaria. Dtsch Med Wochenschr.

[B27] Koh KH, Chew PH, Kiyu A (2004). A retrospective study of malaria infections in an intensive care unit of a general hospital in Malaysia. Singapore Med J.

[B28] Koh KH, Tan CI, Chew PH (2006). Acute renal failure in severe falciparum malaria patients in an intensive care unit in Malaysia. J R Coll Physicians Edinb.

[B29] Trang TT, Phu NH, Vinh H, Hien TT, Cuong BM, Chau TT, Mai NT, Waller DJ, White NJ (1992). Acute renal failure in patients with severe falciparum malaria. Clin Infect Dis.

[B30] Naqvi R, Ahmad E, Akhtar F, Naqvi A, Rizvi A (2003). Outcome in severe acute renal failure associated with malaria. Nephrol Dial Transplant.

[B31] Melzer M (2006). Outpatient treatment of falciparum malaria is possible. BMJ.

[B32] Marsh K, Forster D, Waruiru C, Mwangi I, Winstanley M, Marsh V, Newton C, Winstanley P, Warn P, Peshu N, Pasvol G, Snow R (1995). Indicators of life-threatening malaria in African children. New Engl J Med.

[B33] Dondorp A, Nosten F, Stepniewska K, Day N, White N, South East Asian Quinine Artesunate Malaria Trial (SEAQUAMAT) group (2005). Artesunate versus quinine for treatment of severe falciparum malaria: a randomised trial. Lancet.

[B34] Weatherall DJ, Miller LH, Baruch DI, Marsh K, Doumbo OK, Casals-Pascual C, Roberts DJ (2002). Malaria and the red cell. Hematology Am Soc Hematol Educ Program.

[B35] van Genderen PJJ, van der Meer IM, Consten J, Petit PLC, van Gool T, Overbosch D (2005). Evaluation of plasma lactate as a parameter for disease severity on admission in travellers with Plasmodium falciparum malaria. J Travel Med.

[B36] Svenson JE, MacLean JD, Gyorkos TW, Keystone J (1995). Imported Malaria. Clinical presentation and examination of symptomatic travellers. Arch Intern Med.

[B37] Robinson P, Jenney AW, Tachado M, Yung A, Manitta J, Taylor K, Biggs BA (2001). Imported malaria treated in Melbourne, Australia: epidemiology and clinical features in 246 patients. J Travel Med.

[B38] Jensenius M, Ronning EJ, Blystad H, Bjorneklett A, Hellum KB, Bucher A, Hahaim LL, Myrvang B (1999). Low frequency of complications in imported falciparum malaria: a review of 222 cases in south-eastern Norway. Scand J Infect Dis.

[B39] Matteelli A, Colombini P, Gulletta M, Castelli F, Carosi G (1999). Epidemiological features and case management practices of imported falciparum malaria in northern Italy 1991–1995. Trop Med Intern Health.

[B40] DeVita MA, Bellomo R, Hillman K, Kellum J, Rotondi A, Teres D, Auerbach A, Chen WJ, Duncan K, Kenward G, Bell M, Buist M, Chen J, Bion J, Kirby A, Lighthall G, Ovreveit J, Braithwaite S, Gosbee J, Milbrandt E, Peberdy M, Savitz L, Young L, Galhotra S (2006). Findings of the first consensus conference on medical emergency teams. Crit Care Med.

[B41] Armitage J, Eddleston J, Stokes T (2007). Recognising and responding to acute illness in adults in hospital: summary of NICE guidance. BMJ.

